# A Self-Determination Theory and Acceptance and Commitment Therapy-based intervention aimed at increasing adherence to physical activity

**DOI:** 10.3389/fpsyg.2022.935702

**Published:** 2022-08-16

**Authors:** Dalit Lev Arey, Asaf Blatt, Tomer Gutman

**Affiliations:** ^1^School of Psychology, The Academic College of Tel Aviv-Yaffo, Tel Aviv-Yaffo, Israel; ^2^School of Behavioral Sciences, College of Management Academic Studies, Rishon LeZion, Israel

**Keywords:** Self-Determination Theory (SDT), Acceptance and Commitment Therapy (ACT), exercise adherence, physical activity intervention, motivation to exercise, field study, online intervention

## Abstract

The purpose of the present study was to evaluate the effectiveness of a physical activity (PA) intervention program designed to enhance levels of engagement in PA. Despite robust evidence supporting the beneficial effects of PA on overall health, only about 22% of individuals engage in the recommended minimum amount of PA. Recent surveys suggested that most individuals express intentions to be physically active, though the psychological state of amotivation dismissed these struggles. In the current study, we pilot-tested a new intervention program, aimed at enhancing engagement in PA among sedentary individuals. The intervention was based on two behavioral change and motivational psychological frameworks: Self-Determination Theory (SDT) and Acceptance and Commitment Therapy (ACT). During a 14-week intervention program, 94 sedentary Israeli college students (Mage = 24.4, SD = 1.42, Females = 89) were randomly assigned into one of three groups: SDT and ACT-based intervention, traditional intervention, and a non-treatment group. Prior to and following the intervention, participants completed the Behavioral Regulation in Exercise Questionnaire-3 (BREQ-3) to examine motivation to exercise and the International Physical Activity Measurement IPAQ to evaluate their training frequency. Results showed that the SDT and ACT-based intervention group exhibited a significant increase in motivation to exercise between time 1 and time 2, while the other two groups (i.e., the traditional intervention program and the non-treatment group) showed insignificant differences in motivation to exercise. Furthermore, neither of the groups showed significant differences in their training frequency per week. However, those in the SDT and ACT-based groups reported an increase in activity intensity from time 1 to time 2 compared to the two other groups. Further, exercise psychology consultants and scholars can use the intervention protocol and utilize these findings to improve PA behaviors and promote health in the general population. Limitations, future directions, and implications are discussed in detail.

## Introduction

### Adherence to physical activity

There is a substantial consensus among scientists and health professionals that Physical activity is beneficial for our physical, mental, and cognitive wellbeing (Stoltz et al., 2009; King et al., 2013). Physical activity (PA) is defined as any body movement generated by the contraction of skeletal muscles that raises energy expenditure above the resting metabolic rate and is characterized by its modality, frequency, intensity, duration, and context of practice (Tremblay et al., 2017). Studies demonstrated that physical inactivity doubles health risks and indicate that the disease burden to society caused by physical inactivity is comparable to that caused by smoking and unhealthy eating habits (US Department of Health and Human Services, 2018; World Health Organization, 2019). In contrast, regular PA can help control weight, increase energy levels, improve sleep quality and overall physical health (Hills et al., 2007). Systematic exercise contributes to primary and secondary prevention of several chronic diseases, including cardiovascular disease, diabetes, cancer, obesity, and osteoporosis (Pedersen and Saltin, 2015). Furthermore, among the recognized mental health benefits of PA are improved wellbeing, reduced depression and anxiety, boosted self-confidence, and enhanced cognitive functioning, thereby improving overall quality of life (Hills et al., 2007; Warburton and Bredin, 2017).

Despite robust evidence supporting the beneficial effects of PA on overall health, roughly 22% of individuals in developed nations engage in the minimum amount of PA recommended for public health benefits (Piercy and Troiano, 2018). This index stands for at least 150 min of moderate PA a week or at least 75 min of intense activity a week. In Israel, where the current study took place, the situation is quite similar. According to the most recent survey conducted by the Israel Central Bureau of Statistics (2018) indicated that during 2017, 29.6% of Israel's population aged 21 and above engaged in PA (a cumulative total of at least 30 min during the day) at least three times a week. Males more than females, engage in the recommended amount of PA (31.4 vs. 27.0, respectively). Among the Arab population, as compared to Jews, statistics of PA engagement is even gloomier; only 29.9% of Arab males and 22.7% of Arab females reach the recommended amount of exercise (Israel Central Bureau of Statistics, 2018).

According to international surveys, exercise withdrawal episodes and drop-out rates remain high and have been on the rise in recent years (World Health Organization, 2019). For instance, drop-out rates among new gym members reach the 75% mark after the first three months of membership, and 50% of those who remain drop out after 6 months of membership (Radel et al., 2017). Though a large portion of the world's population does not engage in the recommended levels of PA, recent surveys suggest that the majority express intentions to be physically active (European Union, 2017). This finding points to the potential for increasing PA levels among sedentary populations.

In this regard, recent research has sought to understand what promotes exercise engagement (Rodrigues et al., 2019). According to European Union (2017), the main reasons people cited to justify physical inactivity were “lack of time” and “lack of motivation,” 43 and 23%, respectively. Caudwell and Keatley (2016) claim that both these reasons can be classified under the psychological state of amotivation. The reasons behind this lack of motivation are countless, varying from previous negative experiences, lack of self-efficacy for behavioral change, low social and cultural support, and environmental conditions, such as limited access to exercise facilities and expensive costs of training programs (Teixeira et al., 2012).

### Self-Determination Theory (SDT)

Theories concerning human motivation have long been applied in the PA context. Recently, however, the need for specific theory-driven PA interventions has been highlighted (Malik et al., 2014). One prominent theoretical perspective that appears to be potentially useful for understanding various motivational issues in PA settings is Self-Determination Theory (SDT; Deci and Ryan, 2002). SDT accounts for the quality of motivation-regulating behavior, as well as the processes that facilitate motivational development (Ryan and Deci, 2002). Rooted in humanistic psychology, SDT offers considerable potential for understanding “why” people initiate, persist, and terminate their involvement in various physical activities (Hagger and Chatzisarantis, 2007). In addition, scholars (Ntoumanis, 2001; Teixeira et al., 2012) claim that SDT is the motivational construct most widely used by researchers for understanding the influence of human motivation on behavioral outcomes in the exercise context.

**Figure 1:**
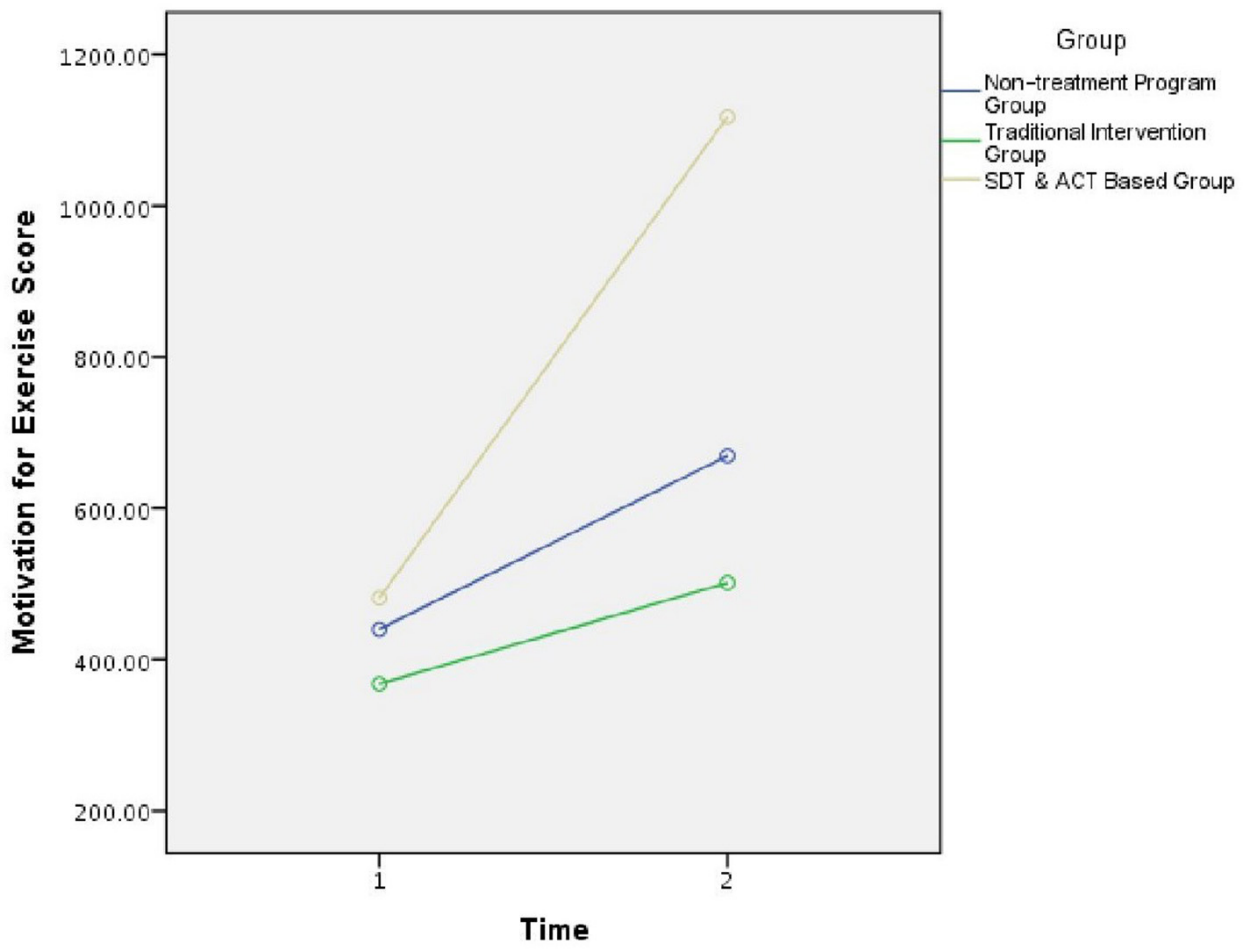
The Self-Determination Theory (SDT) continuum. At one end of the self-determination continuum stands amotivation, at the middle, there are four types of extrinsic motivation, and intrinsic motivation stands on the right at the most self-determined feature of motivation. Autonomy, relatedness, and competence express the basic needs for internal motivation.

SDT postulates that motivation is based on the degree of perceived self-determined behavior. Specifically, Deci and Ryan (1985) proposed several types of behavioral regulation along a motivational continuum (see Figure 1). At one end of the self-determination continuum is *amotivation*, defined as the absence of any type of motivation or lack of intention to act. That is, the individual does not know why to engage in the behavior or does not seek to continue it in the future (Ryan and Deci, 2017). At the other end of the motivational continuum is *intrinsic motivation*. This type of behavioral regulation is the most self-determined form of motivation, according to which individuals act on their own will, based on the experience of pleasure and enjoyment inherent in the behavior (Deci and Ryan, 1985). Between the two poles of the continuum (i.e., amotivation and intrinsic motivation) are four types of *extrinsic motivation* that vary according to the degree of self-determined behavior. (1) External regulation is closest to the amotivation end of the spectrum and explains how behavior is initiated and maintained solely for the sake of external rewards or to satisfy others' needs. (2) Introjected regulation, which is next on the spectrum, still has an external locus of causality but contains a small degree of internalization. Introjected regulation is defined as behavior for the sake of avoiding feelings of guilt related to internal pressures (Deci and Ryan, 2008). (3) Identified regulation is marked by a higher degree of internalization, such that the individual acts due to the innate benefits of the behavior, and it is considered to be a more self-determined component. (4) Finally, integrated regulation, the behavioral regulation most proximal to intrinsic motivation, is defined as the form of motivation that occurs when an individual has fully integrated the behavior (Ryan and Deci, 2017).

SDT can be used to explain individuals' commitment to engaging in PA (Deci and Ryan, 1985). Individuals with autonomous or more self-determined motivational orientation (i.e., intrinsic motivation and the identified and integrated forms of extrinsic motivation regulation) tend to have more favorable attitudes toward PA behaviors. Typically, when PA goals are self-determined, they reflect motivation based on enjoyment (Teixeira et al., 2012), competence, and relatedness (Rodrigues et al., 2019). Previous studies demonstrated that this kind of motivation is correlated with exercise intentions and exercise engagement (Izquierdo-Porrera et al., 2002; Edmunds et al., 2007; Centers for Disease Control, 2008; Prichard and Tiggemann, 2008). When goals are not self-determined, but rather introjected or controlled, they reflect motivation driven by external sources. In general, research findings have demonstrated that this kind of externally driven motivation undermines the development of autonomy and is therefore not optimal for sustaining an intentional PA routine (Ryan and Frederick, 1997; Ryan and Deci, 2000; Segar et al., 2006). Presumably, when individuals feel pressured to exercise, they absent the enjoyment and inner motivation to continue, ultimately causing them to discontinue the behavior.

### Acceptance and Commitment Therapy (ACT)

Interventions to promote sustained PA showed modest success (Van der Bij et al., 2002; Conn et al., 2011). The majority of these interventions rely on cognitive models, such as social cognitive theory (Bandura, 1986), reasoned action approaches (Head and Noar, 2014), and the theory of planned behavior (Ajzen, 1991). In their meta-analysis study, Rhodes and de Bruijn (2013) claimed that the weaknesses of the early models were epitomized by the PA intention-behavior gap. Their results showed that 46% of the people expressed intentions to PA adherence but were unsuccessful in achieving this goal. The theoretical shift began since the early models dismissed internal human motives and self-regulation motives. Herein, an intervention based on a personal connection with guided self-regulation techniques is required to translate PA intention into an actual PA regimen (Rhodes and de Bruijn, 2013).

Hayes (2004) suggested that there are three consecutive waves, representing changes with regards to improving the individual's mental health in terms of cognitive-behavioral therapy framework. Each wave was conceptualized based on the evolution of the previous wave, thus there are multiple common mechanisms among the waves. The first wave focused on the relationship between stimulus and responses, and included characteristics of operant conditioning, reinforcements, and punishments as means of decreasing the intensity of the emotional response. The second wave ascribes great importance to interplay between thoughts-emotions-behaviors and changing dysfunctional cognitions and regulating uncomfortable inner experiences. For example, challenging maladaptive thinking and restructuring the thoughts with more rational thinking patterns. CBT third-wave approaches rely on significant integration between theories and methods such as Eastern philosophy (e.g., Zen approach and Buddhism) alongside Western psychology and the approaches mentioned earlier. The investigation and practice of the third wave approach, which is prevalent today, focuses on the recognition of thought processes and the mode of operation of the mind and consciousness, and not necessarily on the contents of the thought itself; a fuller and happier life will be promoted when the individual is able to accept and contain uncomfortable inner experiences. Out of all the third-wave treatment methods (MBSR, MBCT, DBT, Schema Therapy, and CFT), we found Acceptance and Commitment Therapy (ACT; Hayes et al., 2011) to be the most prominent approach to promote health behavior change (Zhang et al., 2018).

ACT has exhibited positive results in several health domains, such as weight loss (Forman and Butryn, 2015) and smoking cessation (McCallion and Zvolensky, 2015). Through ACT, people may experience new ways of responding to internal events that epitomize the needs for autonomy and competence, which are in accordance with the basic psychological needs to promote internal motivation by SDT. Relying on applied principles from the field of Applied Behavioral Analysis (ABA), previous ACT studies show evidence of individuals being helped to develop and cultivate a new and beneficial behavioral repertoire (Forman and Butryn, 2015). A recent meta-analysis study (Pears and Sutton, 2021) indicated that ACT interventions show promise for increasing PA. Nonetheless, the authors recommended that future ACT interventions to promote PA should name the behavior change processes and techniques used by the ACT matrix.

ACT is based upon acceptance, mindfulness (i.e., acceptance), and value processes (i.e., commitment) to produce psychological flexibility, defined as the ability to take value-based action in the presence of unwanted thoughts, feelings, and bodily sensations (Howell and Passmore, 2019). In line with the ACT matrix, ACT seeks to promote healthy behavioral patterns consistent with stated values while teaching in-the-moment and acceptance skills to increase behavioral commitment to value-based behavior (Lillis and Kendra, 2014; Howell and Passmore, 2019). To deal with the state of amotivation, ACT strategies can increase adherence to exercise goals, which are designed to facilitate the identification of values and create durable commitments consistent with these our values (Levin et al., 2017). Accordingly, an ACT-based intervention aims to strengthen the individual's commitment to behavioral change, build up a willingness to experience a greater range of internal experiences, whether positive or negative, and promote full awareness of exercise behaviors (Butryn et al., 2011).

SDT and ACT-based frameworks share compliable constructs in forms of human motivation and psychological basic needs (Gazla, 2015). In a recent conceptual paper, Ryan (2021), who initiated the SDT framework with Deci and Ryan (1985), argued that the psychological principles within SDT are seen congruent with the third wave framework and value-driven principles expressed by Hayes et al. (1999). Ryan (2021) shared a vision a new movement of a meta-theory that underlies process-oriented assets for mindful awareness, emotional regulation, and autonomous motivation. For instance, boosting psychological resilience, which comprises actions stemming from an individual's inherent values increases internal autonomy in terms of SDT. Hence, it is logical that PA connected to and deriving from an individual's fundamental values assume to contribute to perseverance in engaging in PA over time.

### Purpose and hypotheses

The purpose of the study is to evaluate the effectiveness of a PA intervention program designed to use psychological assets to enhance levels of adherence to exercise. Specifically, the intervention program integrated SDT and ACT-based frameworks, which to our knowledge, this study is the first attempt in the literature to incorporate these frameworks to ameliorate exercise adherence. Our study includes two intervention groups and one control group, namely, the SDT and ACT-based intervention group, a group based on the traditional approach (i.e., internet-based physical activity intervention; Napolitano et al., 2003), and a non-treatment control group. We believe the proposed intervention will ultimately cultivate an inner motivation for change, which in turn will contribute to increasing the frequency and vigor of PA among sedentary population.

#### Hypotheses

H1: Participants in the SDT and ACT-based intervention group will exhibit higher levels of exercise motivation following the intervention, compared to the traditional intervention program group and the non-treatment group (control groups).

H2: Participants in the SDT and ACT-based intervention group will train more frequently per week following the intervention, compared to the traditional intervention program group and the non-treatment group (control groups).

## Methods

### Procedures and participants

Data was collected at the Academic College of Tel Aviv–Yaffo, a mid-size college in the Tel-Aviv (Israel) area. The study was approved by the Ethics Committee of the college. Students were recruited using college bulletin boards, the college Facebook page, and e-mails sent by the college administration. Participants were provided a written description of the study and were invited to a meeting with the research group if they met the following inclusion criteria: (a) 18–65 years of age, (b) in good health that allows them to exercise, (c) willingness to be randomized to type of treatment and start time, (d) have not engaged in exercise for at least 1 year prior to the beginning of the study (i.e., sedentary).

Based on the initial screening, 102 individuals met the inclusion criteria, were judged eligible for the trial and completed the initial evaluation, gave their consent to participate, and were randomized into three groups: SDT and ACT-based intervention, traditional intervention, and a non-treatment group. With the guidance of a professional trainer who was part of the research team, each participant was assigned a personal goal and given a provided with a detailed PA program for the intervention period (14 weeks). The SDT and ACT-based intervention group included 31 participants, three of whom dropped out during the first week, leaving 28 participants in the final group. Mean age for this group was 24.83 (SD = 1.21), and 27 females 96.4%)). The traditional intervention program included 33 participants, four of whom dropped out during the first week, leaving 29 participants. Mean age for this group was 24.38 (SD = 1.66), all 29 were females (100%). The non-treatment group included 38 participants, one of whom dropped out during the first week, leaving 37 participants. Mean age for this group was 24.18 (SD = 1.46), and 36 females 97.3%)). The final number of participants was 94 (see Table 1 for the demographic information of the sample).

**Table 1:** Demographics.

		**SDT and ACT intervention**	**Traditional intervention**	**Non-treatment**
		***N* = 28**	***N* = 29**	***N* = 38**
Gender	Female	27 (96.4%)	29 (100%)	37 (97.4%)
	Male	3 (3.6%)	0	1 (2.6%)
Marital status	Single	11 (39.3%)	18 (62.1%)	18 (47.4%)
	Live w/partner	15 (53.5%)	10 (35.7%)	18 (47.4%)
	Married	1 (3.6%)	0	1 (2.6%)
	Other	1 (2.9%)	1 (3.6%)	1 (2.6%)
Age		24.83 (SD = 1.21)	24.38 (SD = 1.66)	24.18 (SD = 1.46)

### Intervention groups

#### SDT and ACT-based program group

Participants assigned to the SDT and ACT-based exercise intervention group underwent a-14-week program that included theoretical content written by psychologists in accordance with the outline of the proposed program. The researchers initially intended for the meetings to be faced to face, but ultimately the program was delivered *via* Zoom meetings, as the COVID-19 pandemic prohibited any forms of gathering. The protocol of the program was based on intervention methods derived from SDT and ACT frameworks (Ryan and Deci, 2017; see Table 2 for program protocol). During meetings, participants were encouraged to express about their accomplishments and struggles and consult with each other and the professional staff.

**Table 2:** The intervention protocols.

**Module**	**Content**	**Description**	**Theory references**
0	Registration	Publication and invitation among the B.A. students at the “Academic college of Tel-Aviv Yaffo:” “Running Mind—a program for enjoyable and satisfying exercise.”	The need for Autonomy—free choice registration.
1	Introduction and motivational personal investigation	- Introduction to the program schedule and requirements. - Basic motivational intervention—pros and cons of the current and desired exercise status in participants' lives. - Filtering non-ready participants by declaring the option of departure between meeting 1 and 2.	- The need for Autonomy—Setting and personal connection enhancement. - Self as context *via* ACT; reviewing this current status from a curios standpoint
2	Values and values-driven behavior.	- Getting in emotional touch with participants deeper values that motivated them to exercise - Exploration of their internal experience that “get them on their way” to exercise.	- Values and contact with the present moment
3	Thinking traps	- Psycho-Education about the human mind and cognitive traps (for instance, non-adaptive interpretations of PA). - Personal exploration about each participant thinking traps.	Acceptance and the need for relatedness (I'm human like everybody else”).
4	Willingness	Increasing participants' ability to accept their uncomfortable internal experiences while they move toward discovering inherent motives in their PA regimen.	Acceptance, Competence, and value-driven behavior
5	Applied behavioral analysis	- Psycho-Education: long and short terms consequences of PA. - groups discussion about each participant's common behaviors	Committed actions and relatedness
6	Self-compassion	- Psychoeducation and exercises. - Discussion about Self companionate PA. - Personal exploration of SC implementation in each participant's daily PA.	Acceptance and self-compassion
7	Relatedness in action	- Follow up and self-Examination about what is considered productive and unproductive behaviors in current PA status. - Dividing into small support groups.	Self as context and relatedness
8	Summery and relapse prevention	- Psychoeducation about relapse prevention. - “What I have achieved” session and planning for the future.	Competence and committed actions

#### Traditional intervention program group

Participants assigned to the traditional intervention program group participated in the intervention program *via* weekly e-mails over a 14-week period. The information provided was based on the internet-based PA intervention by Napolitano et al. (2003), which has received research support for its success in promoting PA. The program emphasized research-based exercise information in the form of safety tips, staying active, PA and health recommendations, overcoming barriers, planning activity, and benefits of activity. The participants were provided with links to podcasts and workout applications, actual means of contacting local trainers and attending exercise classes. They were also given a worksheet, which included information on how getting started, monitoring progress, setting goals, rewarding oneself and getting support. Participants were accorded with time frames where they could interact *via* ZOOM meeting with registered psychologists and personal trainers.

#### Non-treatment program group

Participants assigned to the waiting list control group were told they would have to wait three months to participate. They completed the same assessment measures at times 1 and 2, similar to the participants in the other interventions groups.

### Study materials

#### Demographic questionnaire

The questionnaire was constructed for the purpose of the study and included the following personal information: age, gender, employment status, marital status, number of children, activities that are not sport related, average household income, number of workouts per week, number of hours of training, and type of favorite exercise.

#### The behavioral regulation in exercise questionnaire-3 (BREQ- 3)

Was used to measure participants' motivation for exercise. The Hebrew version of the measure, used in this study, was created using a double translation method (McGorry, 2000). The BREQ-3 was translated from English into Hebrew by the authors and then back-translated by a professional translator fluent in English and Hebrew. A team of independent judges, made up of psychologists, then considered the equivalence of the original and the back-translated versions of the scales. After discussing instances of non-equivalence, the final version was established. Several studies have provided evidence of the validity of this instrument both in in English version (e.g., Wilson et al., 2022), as well as in other countries and languages such as Italian (e.g., Cavicchiolo et al., 2022), Portuguese (e.g., González-Cutre et al., 2010), and Chinese (e.g., Luo et al., 2022). BREQ-3 measures motivation along the self-determination continuum, based on Self-Determination Theory (Deci and Ryan, 2000). The measure consists of 24 items answered on a 5-point Likert scale and measures the six types of motivation along the SDT continuum, using four items for each motivational score. The questionnaire begins with an overall question: “Why do you engage in exercise?” The six types of motivational regulation tested were: intrinsic regulation (sample item: “I exercise because it's fun”); integrated regulation (sample item: “I consider exercise part of my identity”); identified regulation (sample item: “It's important to me to exercise regularly”); introjected regulation (sample item: “I feel guilty when I don't exercise”); external regulation (sample item: “I exercise because other people say I should”); and amotivation (sample item: “I don't see why I should have to exercise”). Finally, the scales were combined into a single scale, the Relative Autonomy Index (RAI), which measures degree of autonomous regulation as calculated by the following formula:

RAI = (intrinsic regulation ^*^3) + (integrated regulation^*^2) + identified regulation – introjected regulation - (external regulation^*^2) - (amotivation^*^3).

Reliability for the sub-scales was good. Cronbach's alpha for both sessions of the study ranged from 0.72 to 0.96 (see Table 3).

**Table 3:** Cronbach's alpha reliability for the BREQ-3 sub-scales.

**Motivation type**	**Weight**	**First admission**	**Second admission**
Amotivation	−3	0.72	0.71
External regulation	−2	0.91	0.84
Introjected regulation	−1	0.83	0.89
Identified regulation	1	0.81	0.77
Integrated regulation	2	0.94	0.96
Intrinsic regulation	3	0.91	0.93

#### International physical activity measurement (IPAQ)

Participants reported their PA using the long version of the self-report International Physical Activity Questionnaire. We used the cross-cultural adaptation method recommended by the IPAQ committee, which proposes translating the IPAQ into the participants' language, followed by back translation into English by two independent experts. The translated questionnaire was then pilot tested in a convenience sample of 10 individuals. The IPAQ covers five domains (work, transportation, domestic and recreational activities and sitting time) within a time frame of the last seven days. Participants report how many days and how many hours and minutes per day (with a minimum duration of 10 min) they spent engaging in moderate or vigorous activity.

The IPAQ scoring protocol was used to calculate the IPAQ scores. First, a continuous variable of PA was obtained using the Metabolic Equivalent of Task (MET) formula. PA was symbolized by minutes per week, representing the amount of energy expended carrying out certain physical activities. IPAQ defines moderate PA as those that produce a moderate increase in respiration rate, heart rate and sweating for at least 10 min duration. This is equivalent to 3–6 MET based on the compendium of PA. Vigorous physical activities are defined as those producing vigorous increases in respiration rate, heart rate and sweating for at least 10 min duration (Ainsworth et al., 2000). Participants are asked to refer to all domains of PA including occupational, transport, household, yard/garden and leisure/sports. Thus, walking was equivalent to 3.3 METs, moderate PA to 4 METs and vigorous PA to 8 METs. The following formula was used to calculate the MET per week (Forde, 2018):


$$\begin{array}{l} \text{Total}=\;3.3*\text{WalkingNum}*\text{WalkingTime}\\ \;+\text{​ }4*\text{ModerateNum}*\text{ModerateTime}\\ \;+\;8*\text{VigorousNum}*\text{VigorousTime} \end{array}$$

### Data analysis

Repeated Measures ANOVA analysis was used to test the first hypothesis, with time (before and after the program) as an independent variable and group (experiment, control with intervention and audit without intervention) as an independent within- subjects variable. The dependent variable was exercise motivation, as assessed by the BREQ-3. An ANOVA Repeated Measures analysis was conducted to test the second hypothesis, with time (before and after the program) as an independent within-subjects variable and group (experiment, control with intervention and non-intervention audit) as an independent between-subjects variable, while controlling for gender as a covariate variable. The dependent variable was training frequency, as assessed by the IPAQ. Additionally, we conducted repeated measure ANOVA as the dependent variable was represented by the intensity variable.

## Results

**Figure 2:**
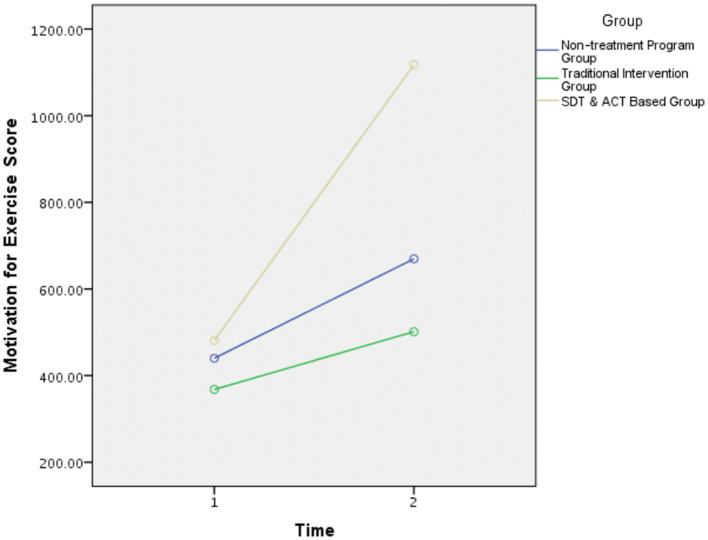
Motivation for exercise score. All groups presented the same level of motivation for exercise score before the intervention (i.e., time 1). Following the intervention (i.e., time 2), SDT and ACT-based intervention group displayed a significant increase in motivation for exercise. Nonetheless, the two control groups (traditional intervention group and non-treatment group) did not show significant differences between the two-time measurements.

The first research hypothesis was that participants in the SDT and ACT-based intervention group would exhibit higher levels of exercise motivation, compared to the traditional intervention program and non-treatment groups. Consistent with the first hypothesis, a significant interaction effect was found, *F*_(2,97)_ = 7.45, *p* < 0.01 (Figure 2). Further analyses of the interaction showed significant evidence of increased motivation for PA in the experimental group after the workshop (M = 58.08, SD = 20.09), compared to before the workshop (M = 39.50, SD = 23.85), *t*_(33)_ = 3.29, *p* < 0.01. In contrast, the two control groups [traditional group, *t*_(37)_ = 0.48, *p* > 0.05, and non-treatment group, *t*_(29)_ = 0.75, *p* > 0.05] did not show significant differences between the two-time measurements. Thus, the first research hypothesis was confirmed.

Moreover, a significant main effect model was found for group, *F*_(2,97)_ = 3.74, *p* = 0.02. Follow-up analysis showed that the overall mean of the experimental group was significantly higher than the general mean of the traditional intervention group, *p* < 0.01, and of the non-treatment control group, *p* < 0.01.

The second hypothesis was that participants in the SDT and ACT-based intervention would increase their training frequencies per week, compared to those in the traditional intervention program and non-treatment groups. The results of the model showed that the interaction effect did not reach significance, *F*_(2,93)_ = 1.53, *p* = 0.22. In contrast, a main effect was found for time, *F*_(1,93)_ = 8.19, *p* < 0.01, in the manner that the PA score after the intervention was higher (M = 1,066.33, SD = 941.87) than prior to the intervention (M = 1,482.74, SD = 1,396.17) in all groups.

**Figure 3:**
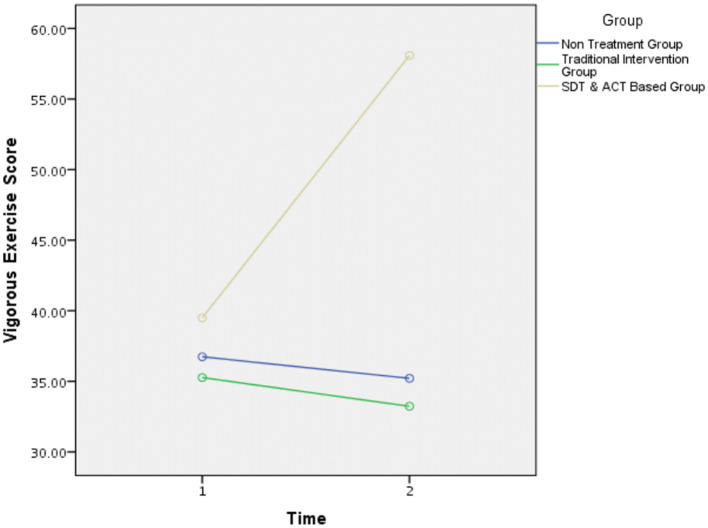
Vigorous exercise score. The three intervention groups displayed a similar level of engagement in intense physical activity before the intervention (i.e., time 1). Following the intervention (i.e., time 2), SDT and ACT-based intervention group demonstrated a significant increase in vigorous exercise score. However, the two control groups (traditional intervention group and non-treatment group) did not show significant differences between the two-time measurements in this index.

Since the interaction effect did not reach significance, a second analysis was conducted. In this analysis, PA was not calculated as a weighted average of walking, moderate activity, and intense activity (overall score), but only as a score representing activity intensity *via* MET. The purpose of this was to examine whether the intervention in the experimental group raised the participants' level of activity intensity compared to the control groups. Examination of this model elicited a significant interaction effect, *F*_(2,98)_ = 3.12, *p* = 0.04. Figure 3 shows the nature of the interaction.

## Discussion

Most intervention programs that aim to promote physical activity as an integral part of life have shown limited effectiveness (Hartmann, 2003). In the present study, we described an intervention based on a firm body of research linked to behavioral change. The first approach, Self-Determination Theory (SDT; Deci and Ryan, 1985), focuses on types of motivation and postulates that intrinsic motivation will make exercise a part of individuals' lifestyle and help them maintain exercise attitudes and persevere in their activities. The second approach, Acceptance and Commitment Therapy (ACT; Hayes et al., 2011), which falls under the umbrella of the third wave of CBT, aims to cultivate the ability to confront one's inner experience (i.e., acceptance) and choose behaviors consistent with one's values (i.e., commitment). This theoretical integration is assumed to increase the efficiency of a specific theory-driven PA intervention (Melkevik et al., 2010), as evidenced by short- and long-term exercise adherence implications.

The present study utilized evidence-based theoretical frameworks to examine motivational barriers that cause individuals to avoid engaging in PA behaviors. The primary framework is based on the distinction SDT makes between internal and external motives. Intrinsic motivation prevents a person from performing an activity for personal interest or enjoyment, while external motivation stimulates a person to perform an activity to gain rewards or prevent losses (Ryan and Deci, 2017). Indeed, when PA goals are self-determined, they align with exercise intention and engagement (Teixeira et al., 2012). The second theoretical framework for mitigating motivational obstacles in our study is ACT (Hayes et al., 2011). This model comprises acceptance, mindfulness, and value processes to produce psychological flexibility, which helps the individual adopt value-based action in the presence of unwanted thoughts, feelings, and bodily sensations. ACT interventions have already shown improvements in health among inactive populations (Forman and Butryn, 2015; Ivanova et al., 2015). The relationship between the core constructs of SDT and ACT received minimal research attention, although these frameworks share analogous assemblies in the forms of human motivation and basic psychological needs (Gazla, 2015). Recently, Ryan (2021) conceptualized these frameworks as a meta-theory that underlies process-oriented properties for mindful awareness, self-regulation processes, and autonomous motivation. We perceive this integration as an innovative and pivotal attempt to promote PA behaviors.

The research hypotheses encompass a desire to engage in exercise (H1) and to display actual exercise behaviors (H2). The study's population included sedentary students attending a mid-size college in Tel-Aviv (Israel), who participated in a 14-week intervention program. The students were randomly allocated to three groups: SDT and ACT-based intervention group, traditional intervention group, and non-treatment group. The research hypothesized that participants in the integrated intervention group would exhibit a greater increase in their level of motivation for PA compared to the two control groups. Moreover, it hypothesized that participants in the SDT and ACT-based intervention group would increase the frequency of their weekly training compared to the two control groups.

The first research hypothesis was supported. The SDT and ACT-based intervention group exhibited a significant increase in motivation to exercise between time 1 and time 2, while the two control groups (i.e., the traditional intervention program and the non-treatment group) showed insignificant differences in exercise motivation. An integrative view of our findings in conjunction with those of previous interventions points to the possibility that receiving autonomy-supportive PA counseling over the course of 3 months increased the participants' motivation for exercise (Fortier et al., 2007). Moreover, an ACT-based intervention among sedentary women has been found to lead to improvements in perceived effort and ratings of post-exercise enjoyment, compared to a control group that engaged in similar tasks, such as the traditional intervention group in our study (Ivanova et al., 2015). Taken together with these findings, the current study offers a solid research direction to explore integrating SDT and ACT to increase motivation for exercise.

The second research hypothesis was partially supported. The groups did not show significant differences in their training frequency per week. Nevertheless, the SDT and ACT-based intervention group exhibited a significant increase in the parameter of activity intensity from time 1 to time 2, while the two control groups showed only insignificant differences in this index. This pattern is also evident in previous studies utilizing ACT techniques (Ivanova et al., 2015) and SDT inclinations (Burn and Niven, 2019) under constant work rate conditions of high intensity. With respect to the non-significant differences in overall training frequency per week, we propose that participants did not assimilate the SDT and ACT assets under low-intensity PA conditions since they perceived these as ordinary activities. This tendency to overlook the contextual features of exercise under conditions of low levels of mental and physical overload may constitute a barrier to implementation of health interventions (Norton and Chambers, 2020). Consistent with this line of thinking, the increment in autonomous behavior may have led to an increased sense of perceived challenge, causing participants to engage in highly intense PA (Ekkekakis, 2009). Nonetheless, the participants in the SDT and ACT-based intervention increased their PA regimen compared with those in the control groups.

The research hypotheses were corroborated. Indeed, it seems that the unique intervention program enhanced participants' level of motivation and increased their frequency in intense training per week. To our knowledge, the 14-week intervention program proposed in this study is the first attempt in the literature to incorporate the SDT and ACT frameworks to ameliorate exercise adherence. Exercise consultants and scholars can utilize out intervention protocol and these findings to promote health in the general population.

### Limitations, future directions, and implications

While these findings provide evidence for the effectiveness of a specific theory-driven PA intervention, one should be aware of the potential limitations of this study. The first pertains to the sample, which included primarily white female participants from one specific college. Future research is needed to evaluate the program with a more diverse sample, using male participants, different populations, and minority groups. Furthermore, the ACT matrix includes six core processes for enhancing clients' psychological flexibility: being present, acceptance, cognitive defusion, self as context, values, and committed action (Ivanova et al., 2015). The first component, being present, represents mindfulness features. According to Tang et al. (2016), studies that encompass such concepts should also measure differences in dispositional mindfulness (i.e., trait mindfulness) among participants to exclude confounding factors.

Another limitation is related to procedural issues. The intervention study was conducted between April and June 2021. During that period, COVID-19 spread rapidly in Israel, and the government declared a lockdown. People were instructed to remain within 100 m of home, and all social services were shut down, including, schools, academic institutions, and gyms. A study that examined the implications of the lockdown for Israelis found that about 48% of the public exhibited negative emotional reactions, including feelings of threat, shock, and chaos at home (Levkovich and Shinan-Altman, 2021). Based on these findings, it is likely that motivation to exercise was temporarily negatively affected, at least among some of the participants. In addition, due to the physical distancing requirements and the closure of academic institutions, the workshop meetings and working group meetings took place virtually *via* the Zoom software. Although this software provides partial consolation for physical distancing and isolation challenges, some people found Zoom to be a barrier to learning (Gray et al., 2020). Furthermore, technical issues such as internet connection issues may affect learning. Considering these issues with Zoom along with limited access to exercise facilities, it may be worthwhile to examine the effectiveness of the program when frontal sessions are no longer prohibited in order to reinforce the findings of the present study.

The study also has some conceptual limitations. It relied primarily on a self-report motivation questionnaire (BREQ-3) and an exercise questionnaire (IPAQ). These self-report instruments introduce substantial bias when assessing exercise behaviors. For instance, several studies have reported that the IPAQ may tend to overestimate the extent of overall PA (Dyrstad et al., 2014). Therefore, the effect of the program should also be examined using objective metrics such as weight loss and muscle mass gain or other objective metrics tailored to the goals set for each participant at the beginning of the study. Moreover, studies indicate that most dropout from exercise programs occurs during the first 6 months (Vojvodic et al., 2020). The current study did not include a long-term follow-up assessment. Future studies should examine this factor.

This study provides further evidence that an integrated intervention program oriented toward SDT and ACT leads to improved health outcomes. It is possible that SDT increased the participants' motivational attitudes and that ACT augmented their psychological flexibility. Yet the participants did not necessarily transform their exercise regimen based on these properties. One alternative intervention would be to illustrate the delivery of consulting techniques under physical conditions that simulate exercise situations. For example, a previous study used a task that entailed placing hands into a bowl of ice cubes (e.g., Ivanova et al., 2015) to practice cognitive diffusion conceptualized by the ACT matrix. Alternatively, future studies should use SDT and ACT component measures to validate the pathway influences of each framework. For example, assimilating the Acceptance and Action Questionnaire-2 (AAQ-2; Bond et al., 2011) in a future study to evaluate the psychological flexibility index.

The population of the study consists of young adults. Although this population is perceived as healthy, <27% of students described their lifestyle to be active (Yahia et al., 2016), and reduced PA levels were observed among this population due to the Covid-19 pandemic (Gallo et al., 2020). Previous studies showed that SDT improved PA rates among students (Jenssen and Dillern, 2021) likewise ACT (Wang et al., 2020). Specifically, young adults may gain mental and physical advantages by assimilating the protocol of the SDT and ACT-based intervention group and facilitates PA adherence throughout their professional career.

To conclude, this intervention study exhibited significant and positive results regarding the effectiveness of an integrated SDT and ACT-based intervention aimed at increasing exercise engagement. Considering human motivation aspects, such as autonomy, competence, and value-based actions to encourage a sedentary population to sustain PA behaviors and make it an integral part of their lives. Moreover, we believe that exercise psychology practitioners can use our intervention protocol and the knowledge extracted from this study to guide their clients with the aim of physical and mental health promotion.

### “Running minds” intervention

The intervention group participated in eight (90 min each) workshops, followed by a new integrative protocol, developed to encourage people to experience appetitive and enjoyable exercises in their daily lives. It leans on Gardner and Moore (2007) Protocol (*The psychology of enhancing human performance: The mindfulness-acceptance- commitment (MAC) approach*), Functional Behavioral Analyses, Motivational Interviewing, and SDT basic needs perspective. The protocol contains eight modules, including (0) registration (1) introduction and motivational personal investigation; (2) values and values-driven behavior; (3) thinking traps; (4) Willingness; (5) applied behavioral analysis in FA; (6) compassionate exercises principles, (7) relatedness in action, (8) summary and relapse prevention. The first four workshops were delivered every week, and the last four were delivered every 2 weeks. First, we published the course among the B.A. students at the “Academic College of Tel-Aviv Jaffa,” under the title—“Running Mind—for enjoyable and satisfying exercise” and invited those who registered for an introduction workshop. In order to create a homogenous working group, we asked participants to fill in a short questionnaire about their current PA status. Then, we divided them into three groups—not active students, moderate active students (2 PA per week), and high active students (3 or more PA per week). Each module began with a brief psycho-education and Q and A. Then, participants were divided into smaller groups to encourage an open discussion and a personal exploration, guided by a physical trainer with B.A. in Psychology.

## Ethics statement

The studies involving human participants were reviewed and approved by the Academic College of Tel Aviv-Yaffo, Tel Aviv-Yaffo, Israel. The patients/participants provided their written informed consent to participate in this study.

## Author contributions

DL designed the study, collected and analyzed the data, and wrote the manuscript. AB executed crucial edits in the study and wrote the manuscript. TG wrote the program protocol and guided the intervention program. All authors actively contributed to writing the manuscript. All authors contributed to the article and approved the submitted version.

## Funding

The authors wish to express their gratitude to the Research Fund of the Research Authority, College of Tel Aviv-Yaffo, Tel Aviv-Yaffo, Israel, for the financial support provided for this publication.

## Conflict of interest

The authors declare that the research was conducted in the absence of any commercial or financial relationships that could be construed as a potential conflict of interest.

## Publisher's note

All claims expressed in this article are solely those of the authors and do not necessarily represent those of their affiliated organizations, or those of the publisher, the editors and the reviewers. Any product that may be evaluated in this article, or claim that may be made by its manufacturer, is not guaranteed or endorsed by the publisher.
