# Supplementary material for: A Self-Determination Theory and Acceptance and Commitment Therapy-based intervention aimed at increasing adherence to physical activity
Source: Front Psychol. 2022 Aug 16;13:935702. doi: 10.3389/fpsyg.2022.935702 (PMC9426339; doi:10.3389/fpsyg.2022.935702)
Supplement: Supplementary file 1 [file Data_Sheet_1.PDF]

Example of information provided to Traditional Intervention group

Week 1 – The importance of Physical activity for our physical, mental and cognitive health, general information

Hello,

Welcome to an Healthy Lifestyle and Exercise study!

We thank you for choosing to participate in our research, and happy that you will take part in your process.

This experience may be a turning point in your life if you are open-minded, ready, and willing to experiment, learn and develop.

We compiled for you various professional materials that deals with a healthy lifestyle and physical activity. During the span of the research, you will receive a weekly email that will include content on various topics related to physical activity and its impact on your health and life. We hope you enjoy the process and get the most out of it.

So, let's get started!

The first topic we will focus on is: Why is it important for you to engage in physical activity? What are the benefits? Why in practice is it beneficial to us? What happens to our body during activity? (Both physically and mentally).

Here are 5 reasons why it is important to be physically active:

1. Contributes to brain activity – Physical activity helps us hone our senses, feel better, and of course maintain proper and sharp brain activity. For example, it improves our reaction speed, speed of thought, reflexes, and muscle response.
2. Improves our mental state - The mind and the body are not two separate entities—although they are often treated that way. Physical health and emotional health are intimately intertwined in what's known as the mind-body connection. Research shows the obvious improvement in our mental health when we take care of our bodies (e.g., eat well, sleep, engage in physical activity), and vice versa.
3. Helps the immune system - People who regularly engage in PA, usually feel stronger and healthier than those who don't. Activity will lead to a healthier body, strong muscles, a strong mind and the prevention or decreased chances of common diseases, such as types of cancer, heart problems, diabetes and more.
   Here is a short movie you might want to watch:
   https://www.youtube.com/watch?v=KEhbYNmY3N4&feature=emb_title
4. Relieves stress - Most individuals experience much stress throughout their lives, due to the various conflicting commitments that we have (e.g., home, work, children, chores). Regular aerobic exercise will bring remarkable changes to your body, your metabolism, your heart, and your spirits. It has a unique capacity to exhilarate and relax, to provide stimulation and calm, to counter depression and dissipate stress. It's a common experience among

endurance athletes and has been verified in clinical trials that have successfully used PA to treat anxiety disorders and clinical depression.

5. Anti-Aging Effect - Research shows that exercise's powerful impact on our physical and mental health can in fact slow down the aging process. This is due to: (a) Increase energy efficiency - As we age, if we are active, we maintain our 'horsepower,' or fuel economy. This suggests that those who exercise can maintain a better quality of life because of their ability to move around easily. (b) Makes our skin younger, by increasing blood flow, sending more oxygen and eliminating waste, exercise keeps skin cells healthy and vital. (c) Improves posture. Due to muscle loss and bone density changes as you age, your ability to keep a healthy posture starts to decline. By strength training you can rebuild muscle and prevent bone loss. (d) Improves flexibility. Although any type of exercise, including both aerobic and anaerobic exercise, can improve our flexibility, yoga and Pilates in particular are highly effective at increasing flexibility as we age.

If you want to understand and see a little more about what happens to us inside our body when we are physically active, we recommend watching the following video - https://www.youtube.com/watch?v=wWGulLAa0O0&ab_channel=BritishHeartFoundation

Enjoy watching and reading! See you again next week, where we will offer additional resources on the benefits od PA, along with address, web pages, phone numbers and references for PA professionals. We will also provide you with several helpful applications for working out, with the hope that this will assist you in igniting your PA rutting.

—----------

Additional References regarding the benefits of PA:

https://personal-fit.co.il/20-%D7%A1%D7%99%D7%91%D7%95%D7%AA-%D7%9C%D7%9E%D7%94-%D7%97%D7%A9%D7%95%D7%91-%D7%9C%D7%A2%D7%A9%D7%95%D7%AA-%D7%A1%D7%A4%D7%95%D7%A8%D7%98/

https://www.youtube.com/watch?v=BHY0FxzoKZE&ab_channel=TED

https://aguda.co.il/%D7%A7%D7%A9%D7%A8-%D7%91%D7%99%D7%9F-%D7%A4%D7%A2%D7%99%D7%9C%D7%95%D7%AA-%D7%92%D7%95%D7%A4%D7%A0%D7%99%D7%AA-%D7%9C%D7%91%D7%99%D7%9F-%D7%91%D7%A8%D7%99%D7%90%D7%95%D7%AA-%D7%A0%D7%A4%D7%A9%D7%99/?gclid=Cj0KCQjw_4-SBhCgARIsAAlegrULczMPIN23adDWvLU5gAPPJHUi7oWV3RpgPcSqjKCmzQM1I54NgjEaAgkgEALw_wcB

https://www.mishan.co.il/good_to_know/%D7%A4%D7%A2%D7%99%D7%9C%D7%95%D7%AA-%D7%92%D7%95%D7%A4%D7%A0%D7%99%D7%AA-%D7%95%D7%94%D7%A9%D7%A4%D7%A2%D7%AA%D7%94-%D7%A2%D7%9C-%D7%94%D7%9E%D7%A6%D7%91-%D7%94%D7%A0%D7%A4%D7%A9%D7%99/
